# Extracellular vesicles in Inflammatory Skin Disorders: from Pathophysiology to Treatment

**DOI:** 10.7150/thno.45488

**Published:** 2020-08-07

**Authors:** Shuai Shao, Hui Fang, Qingyang Li, Gang Wang

**Affiliations:** Department of Dermatology, Xijing hospital, Fourth Military Medical University, Xi'an, Shannxi, 710032, China.

**Keywords:** extracellular vesicle, inflammatory skin disorders, biomarker, therapy

## Abstract

Extracellular vesicles (EVs), naturally secreted by almost all known cell types into extracellular space, can transfer their bioactive cargos of nucleic acids and proteins to recipient cells, mediating cell-cell communication. Thus, they participate in many pathogenic processes including immune regulation, cell proliferation and differentiation, cell death, angiogenesis, among others. Cumulative evidence has shown the important regulatory effects of EVs on the initiation and progression of inflammation, autoimmunity, and cancer. In dermatology, recent studies indicate that EVs play key immunomodulatory roles in inflammatory skin disorders, including psoriasis, atopic dermatitis, lichen planus, bullous pemphigoid, systemic lupus erythematosus, and wound healing. Importantly, EVs can be used as biomarkers of pathophysiological states and/or therapeutic agents, both as carriers of drugs or even as a drug by themselves. In this review, we will summarize current research advances of EVs from different cells and their implications in inflammatory skin disorders, and further discuss their future applications, updated techniques, and challenges in clinical translational medicine.

## Introduction

Extracellular vesicles (EVs) are cell membrane-derived phospholipid bilayer structures that can be produced and released by almost all cell types and range in diameter from 30 to 2000 nm 1. EVs are found in various biofluids and tissues such as serum, milk, urine, and blister, to name just a few 2. Currently, EVs are highly heterogeneous and can be classified into three different subtypes based on their size and biogenesis pathway: exosomes (30-150 nm) which are formed by the inward budding of the endosomal membrane during the maturation of multivesicular endosomes, and microvesicles (MVs)/microparticles (100-1500 nm) or apoptotic bodies (500-2000 nm) that are pinched off from the plasma membrane. However, these EV subtypes display overlapping sizes, compositions and densities. Once released into the extracellular space, EVs can be taken up by recipient cells via activation of surface receptors, vesicle internalization (endocytosis), or fusion with target cells to shuttle biological information including various RNAs, lipids and proteins between cells 3. Thus, EVs regulate multiple biological and pathophysiological processes, including immune responses 4, antibacterial activities 5, cell proliferation and migration 6, and angiogenesis 7, 8, among others, suggesting their pathogenic roles in inflammation progression and cancer metastasis.

Multiple methods have been developed to isolate EVs, including density gradient centrifugation, filtration-based methods and affinity-based or precipitation-based methods. Among them, a density gradient combined with ultracentrifugation is the most widely used and highly recommended method, with low yield and high purity 9. Regarding identification methods, the International Society of Extracellular Vesicles has released guidelines that include transmission electron microscopy (TEM), NanoSight particle size analysis, dynamic light scattering, and the identification of protein markers by western blotting or flow cytometry 10. In some studies, researchers used mass spectrometry to analyze EV proteins and quantify their relative abundances or RNA-sequencing (RNA-seq) to analyze the expression of non-coding RNAs, for example, microRNAs (miRNAs), long non-coding RNAs (lncRNAs), or circular RNA (cirRNAs). EV cargoes reflect their cellular origin and surrounding environmental stimuli.

Recent work from us and others has indicated that EVs play key immunomodulatory roles in the pathogenesis of various inflammatory skin diseases, such as psoriasis 11, 12, atopic dermatitis (AD) 13, lichen planus (LP) 14, bullous pemphigoid (BP) 15, systemic lupus erythematosus (SLE) 16, and chronic wound healing 7. These inflammatory skin disorders pose major problems in dermatology given their complex pathophysiology and refractory nature, which ultimately pose a burden to the health, economic and social systems. As the level of EVs or their cargoes in body fluids may differ between patients and healthy controls, EVs have been used as potential biomarkers of inflammatory skin disorders 17-19. More importantly, EVs are regarded as ideal therapeutic agents in addition to their native bioactivities, and they can be engineered to deliver a variety of proteins, nucleic acids and/or chemicals or drugs. Here, we discuss the current knowledge on the specificities and regulatory functions of EVs (mainly referring to exosomes and MVs) derived from immune and non-immune cells, their roles in the pathogenesis and treatment of inflammatory skin disorders, and the challenges ahead.

## Characteristics and functions of EVs from cells associated with cutaneous inflammation

EVs can be secreted by immune or non-immune cells and affect both innate and adaptive immunity, including antigen presentation, cell differentiation and activation, and immune regulation and suppression, among others. In general, EV surface markers and cargo contents as well as the functions of these EVs are closely related to the pro-inflammatory or anti-inflammatory properties of the parent cells, as summarized in **Table 1.** Here, we mainly discuss the functions of EVs from cells associated with cutaneous inflammation.

### Immune cell-derived EVs

#### Dendritic cell (DC)-derived EVs

DCs are the most efficient antigen-presenting cells that take up, process and present antigens to T cells, which contribute to the initiation and progression of immune responses 20. Early studies reported that DC-derived EVs expressed high levels of functional major histocompatibility complex (MHC) class I (MHC-I) and class II (MHC-II), MHC-peptide complexes, T cell costimulatory molecules, and tumor antigens 21-23. In addition, it was shown that EVs derived from LPS-induced DCs carried multiple classes of RNAs, including miRNAs, small nucleolar RNAs, Y-RNAs, tRNAs, and small nuclear RNAs 24, which may have roles in balancing T helper 1(Th1)/Th2 responses 25.

DC-EVs have been reported to exert either inhibitory or -stimulatory effects on the immune system, depending on the maturity of the originating cell and the subpopulations of EVs. For instance, EVs from mature DCs carried more MHC-II and costimulatory molecules, which was critical for priming T cell responses and activating the immune system 26-28. In a murine heart transplant model, donor mature DCs released EVs that transferred MHC molecules to recipient conventional DCs, ultimately initiating alloreactive T cell responses and acute rejection 29. In contrast, in a rat model of liver transplant, EVs derived from immature DCs induced graft tolerance 30. Moreover, the antigen presenting capacities of EVs from DCs varied among EV subtypes. For instance, compared to MVs derived from mature DCs, small EVs (sEVs) were much more efficient in inducing antigen-specific CD8^+^ T cells and eliciting antigen-specific immunoglobulin G (IgG) production 31. Similarly, sEVs derived from immature DCs promoted the secretion of Th1 cytokines, such as interferon-γ (IFN-γ), while larger EVs (lEVs) induced Th2 cytokine secretion 32. Other functional proteins, such as toll-like receptor 4 (TLR4), could be transferred between DCs through EVs, which increased cellular responsiveness to lipopolysaccharide (LPS)-induced inflammation in recipient cells 33. In addition, CD301b^+^ perivascular DCs continuously released EVs carrying circulating antigens and allergens to neighboring mast cells, and the latter could vigorously degranulate and trigger anaphylaxis 34. DC-EVs were also responsible for viral evasion and deposition, as DC-EVs may harbor viral components and transfer infection-associated factors in hepatitis C virus, dengue virus, and human immunodeficiency virus infection 35-37.

#### B cell-derived EVs

B cells are critical modulators of innate and adaptive immune responses as they participate in antigen-specific interactions and antibody production 38. Similarly, several early studies shown that B cell-EVs expressed a multitude of proteins including MHC-I and MHC-II molecules 39, 40, costimulatory and adhesion molecules, C3 fragments 41, CD20, CD45, heat shock proteins, and pyruvate kinase type M2 40, 42, which are crucial for antigen presentation and T cell responses. Interestingly, stimulation of receptors on B cells, such as CD40 and IL-4 receptor, or B cell receptor (BCR) cross-linking, could enhance the secretion of EVs 43, 44.

Recently, the functions of B cell-EVs in the tumor microenvironment and inflammation have attracted much attention. EVs derived from CD40/IL-4-induced B cells could be loaded with miRNA-155 mimics or inhibitors using the electroporation method and transferred to macrophage cell lines 45, thus reducing the expression of endogenous miRNAs in recipient cells, which suggested that B cell-EVs are an efficient delivery strategy. Klinker et al. reported that MHC-II^+^FasL^+^ EVs isolated from B cell-derived lymphoblastoid cell lines could induce antigen-specific apoptosis in autologous CD4^+^ T cells, suggesting that this kind of EVs had immunosuppressive functions 46. Although B cells and derived autoantibodies are crucial for the progression of cutaneous autoimmune diseases, including BP 29, 47 and SLE 48, the roles of B cell-EVs in dermatology are largely unknown and warrant further study.

#### T cell-derived EVs

T cells are key regulators of the adaptive immune responses involved in inflammatory and autoimmune skin disorders. T cell receptor (TCR) activation and intracellular calcium stimulation could increase EV secretion 49. It has been shown that T cell-derived EVs contained MHC- I and -II, TCR, CD3, APO2 ligand, adhesion molecules such as CD2 and LFA-1, FasL, and chemokine receptors, among others 49, 50. These bioactive T cell-EVs could be taken up by different cell types, inducing a variety of immunoregulatory effects, such as inhibiting NK cell cytotoxicity 51, regulating DC maturation 52, and enhancing B cell responses and antibody production 4, 53.

As T cells are classified into several subsets, their EVs exert distinct effects. It was previously reported that EVs released from CD4^+^T cell subsets, including Th1, Th17 and Treg cells, contained different patterns of miRNAs, suggesting distinct functions for T cell subset-derived EVs 54. Okoye et al. showed that Treg cell-EVs transferred Let-7d to Th1 cells to inhibit their proliferation and IFN-γ secretion, thus contributing to the suppression and prevention of systemic immune diseases 55. Activated CD8^+^ T cells not only prevented tumor progression by direct cytotoxicity against tumor cells but also released EVs to induce mesenchymal tumor stromal cell apoptosis 56. Moreover, another study revealed that T cell-EVs could stimulate human mast cells to degranulate and release several cytokines, such as IL-24, which in turn activated keratinocytes* in vitro* 57. Increasing evidence has suggested that EVs also participated in T-B cognate interactions. T cell-EVs or their delivered miRNAs/lipids regulated B cell survival, proliferation, and antibody production 4, 53, 58, suggesting that T cell-EVs could be engineered to treat B cell overactivation-related diseases.

#### Macrophage-derived EVs

Macrophages are a highly heterogeneous population that can be activated to differentiate into different phenotypes, including pro-inflammatory M1 or anti-inflammatory M2 macrophages. Macrophages release EVs containing mRNAs that help to identify the phenotype of the parent cells 59. Using mass spectrometry, the contents of EVs derived from macrophages that were exposed to LPS were identified and included groups of functional proteins, such as plasma membrane-associated proteins, chaperones, metabolic enzymes, cytokines, alarmins, and damage-associated molecular patterns 60-62. It was also shown that macrophage-EVs were enriched in the active accessible pool of cholesterol, which increased the efficiency of cholesterol transfer to high-density lipoprotein, thus lowering the cholesterol level 63. A recent study reported that Wnt proteins, such as Wnt3a and Wnt7b, were enriched in macrophage-EVs, thus activating the Wnt/β-catenin signaling pathway to enhance hair follicle growth *in vivo* and *in vitro* 64.

On the other hand, macrophage-EVs play a pro-inflammatory role in chronic inflammation and metabolic reprogramming. For instance, macrophage-EVs could induce naive monocyte differentiation via transferring miR-223, which regulated host defense and inflammation 65. EVs released from hemorrhagic shock-activated macrophage promoted reactive oxygen species (ROS) production in neutrophils and their subsequent necroptosis 66. In addition, macrophage-EVs activated corresponding recipient cells such as fibroblasts 67, hepatocytes 68, or epithelial cells 69, which contributed to the progression of cardiac injuries, liver injuries, or acute lung injuries. Moreover, once EVs derived from DNA damage-induced macrophages were taken up by recipient cells *in vivo*, the enhanced cellular glucose uptake and metabolic reprogramming in recipient cells triggered pro-inflammatory responses that augmented chronic inflammation 70. On the other hand, macrophage-EVs are critical for some anti-inflammatory reactions. For example, in a diabetic skin wound healing rat model, macrophage-EVs inhibited the secretion of pro-inflammatory mediators, thus inhibiting inflammation and accelerating diabetic wound healing 71. In addition, macrophage-EVs negatively regulated endothelial cell migration by facilitating the internalization and proteolytic degradation of surface integrin β1 72. As macrophages are one of the key regulatory cell populations in the context of chronic inflammation 73, the role of macrophage-EVs in psoriasis, AD, and skin regenerating needs to be further elucidated to advance the field.

#### Neutrophil-derived EVs

Neutrophils are one of the most critical innate immune cells that exert antimicrobial effects via phagocytosis, degranulation, and neutrophil extracellular traps (NETs). Though short-lived, neutrophils can modulate the immune responses in inflammation and cancers 74. Neutrophil-EVs, first identified by Stein and Luzio 75, were reported to be increased in the circulation during sepsis and inflammation 76. In response to diverse activators, neutrophils generate EVs with different contents such as neutrophil-associated receptors (CD11b, CD18, CD62L, Fc receptors, and complement receptors) and granule proteins (myeloperoxidase, lactoferrin, elastase, matrix metallopeptidase 9, proteinase 3, heat shock proteins, and S100 calcium-binding protein A8) 77-84. One recent study showed that 22 miRNAs and 281 lncRNAs were dysregulated in neutrophil-EVs from patients with diffuse cutaneous systemic sclerosis, suggesting a potential role for EVs in the diagnosis and treatment of autoimmune diseases 85.

As neutrophils are indispensable for controlling bacterial and fungal infections, their EVs contribute to the defense against pathogens in several ways. It has been found that neutrophil-EVs were enriched in antimicrobial proteins, which could inhibit the growth and reproduction of bacteria or fungi 76, 86. Neutrophil-EVs also shuttled arachidonic acid into platelets, and these activated platelets in turn elicited a full neutrophil response, ultimately facilitating neutrophil influx into the lung to eliminate infections 87. Interestingly, EVs released from *Mycobacterium tuberculosis*-infected neutrophils could induce autophagy and superoxide anion production in macrophages, thus indirectly promoting the clearance of intracellular *Mycobacterium tuberculosis* 88.

In addition, neutrophil-EVs can modulate the pro- and/or anti-inflammatory responses of target cells. For instance, neutrophil-EVs enhanced the expression of pro-inflammatory molecules in endothelial cells 89, 90. Neutrophil-EVs containing neutrophil elastase degraded the extracellular matrix, which exacerbated chronic obstructive pulmonary disease 91. Moreover, we showed that neutrophil-EVs activated adjacent keratinocytes and increased the expression and production of pro-inflammatory mediators, which induced a vicious cycle in severe pustular psoriasis 82. In addition, neutrophil-EVs exerted anti-inflammatory effects, such as reducing pro-inflammatory mediators to protect the cartilage 92, inhibiting the production of pro-inflammatory cytokines and enhancing anti-inflammatory cytokines in NK cells 93, monocyte-derived DCs 94, or macrophages 95-97, which indirectly limited excessive inflammatory responses. Neutrophil-EVs from joint aspirates of gouty arthritis patients had similar anti-inflammatory properties. In response to C5a, neutrophils released phosphatidylserine-positive EVs to suppress the inflammasome activation that was primed by C5a and consequently inhibited IL-1β release and neutrophil influx 98.

Furthermore, neutrophil-EVs also modulate endothelial permeability and vascular remodeling in a cargo-dependent manner. Neutrophil-EVs were able to disrupt junctional integrity and increase permeability due to the activities of S100A8, S100A9, myeloperoxidase (MPO), and cathepsin G, among others 81, 99, 100-102. In contrast, neutrophil-EVs transferring barrier-protecting proteins, such as annexin 1, maintained junctional integrity and decreased permeability 103, 104. Thus, it is worth further exploring the regulatory role of neutrophil-EVs in inflammatory skin diseases that display vascular remodeling in skin lesions.

### Non-immune cell-derived EVs

#### Mesenchymal stem cell-derived EVs

Mesenchymal stem/stromal cells (MSCs) are self-renewing, multipotent stromal cells that can differentiate into a variety of cell types. MSCs mainly exert immunomodulatory effects associated with tissue homeostasis and regeneration. MSC-derived EVs are reported to have multiple biological functions, including anti-inflammation, tissue repair, immunosuppression, and neuroprotection 105. Thus, recent studies have focused on exploiting MSCs-EVs as a possible noncellular therapy, which will be discussed in Section 4 of this review. RNA-seq studies showed that MSC-EVs were enriched for distinct classes of RNAs 106-108, and the proteomics profiling of MSC-EVs identified functional proteins involved in cell proliferation and apoptosis, inflammation, extracellular matrix remodeling, and angiogenesis 109-112.

In general, MSC-EVs mainly play protective roles in inflammation. For instance, in tubular or renal injury, MSC-EVs regulated proliferative or anti-apoptotic pathways in tubular epithelial cells 113, 114, shuttled several pro-angiogenic transcription factors 115, or suppressed CX3CL1 expression and reduced the subsequent infiltration of macrophages in the damaged kidney 116. Recent* in vivo* studies further indicated that MSC-EVs promoted the recovery of kidney function in animal models of ischemia-reperfusion-induced acute kidney injury, which could be considered as a future potential therapy 117. Moreover, some studies have reported that MSC-EVs could be used for the treatment of liver diseases, as MSC-EVs suppressed the epithelial-to-mesenchymal transition 118, increased hepatocyte regeneration 119, and decreased the proliferation of hepatic stellate cells 120. They also reduced the level of serum alanine aminotransferase and pro-inflammatory cytokines in liver injury mice, suggesting that MSC-EVs have anti-inflammatory effects in liver injury 121. Human umbilical cord MSC-EVs carrying circular RNAs could inhibit ischemia-induced pyroptosis and the release of downstream IL-1β and IL-18, which helped repair ischemic muscle injury 122.

In dermatology, adipose tissue-derived MSC-EVs attenuated pathological symptoms in an AD mouse model, reducing clinical scores levels of IgE and eosinophils in the blood, the infiltration of immune cells in skin lesions, and the mRNA expression of various inflammatory cytokines 123, which indicated that MSC-EVs could be a novel and promising therapeutic strategy for AD treatment.

#### Keratinocyte-derived EVs

Keratinocytes, the main components of the epidermis, can sense pathogens and mediate immune responses. Dysregulation of keratinocytes and their crosstalk with other immune cells gives rise to the initiation and propagation of inflammatory skin diseases in susceptible individuals 124. Similar to EVs derived from other cells, keratinocyte-EVs also vary in composition and abundance of contents depending on the parent cell status and stimulus. For example, IL-17A-treated keratinocytes released EVs containing β-defensin 2 and chemoattractants such as CXCL1, CXCL3, CXCL5, and CXCL6 125. In addition, *Staphylococcus aureus* (*S. aureus*) enterotoxin B-loaded HaCaT cells (a keratinocyte cell line) released EVs containing MHC molecules, which promoted CD4^+^ and CD8^+^ T cell proliferation *in vitro* 126. Keratinocyte-EVs were also reported to carry a set of miRNAs that helped discriminate between EV subpopulations 127.

Keratinocytes-EVs are actively involved in cellular cross-talk, thus regulating various functions associated with skin homeostasis, including wound healing, proliferation, and pigment production. For example, keratinocyte-EVs carried cathepsin B, transforming growth factor binding protein, and matrix metalloproteinase-1, which stimulated fibroblasts to facilitate extracellular matrix remodeling and subsequent keratinocyte migration during wound healing 128, 129. In skin pigmentation, EVs from ultraviolet B-irradiated keratinocytes significantly increased both the expression and activity of melanosomal proteins in melanocytes 130, partially explaining how the cell crosstalk regulated pigmentation. In addition, EVs derived from keratinocyte induced by IFN-γ could be internalized by bone marrow-derived cells, and the latter cells differentiated into a mature phenotype with enhanced CD40 expression and increased IL-6, IL-10, and IL-12 production 131. Given that keratinocytes play vital roles in host defense and immune responses in skin lesions, it is hypothesized that their EVs may act as regulators in the complicated immune disorders of inflammatory skin diseases.

#### Fibroblast-derived EVs

Fibroblasts can synthesize the major components of the extracellular matrix in connective tissue, thus maintaining the structural integrity of most tissues. Increasing evidence indicated that fibroblasts and their derived EVs exhibited functional specializations according to their source organ and spatial location 132. For instance, cardiac fibroblasts-EVs could transfer miR-21-3p to cardiomyocytes and induce cardiomyocyte hypertrophy 133. Fibroblast-EVs from patients with eosinophilic asthma promoted epithelial cell proliferation, thus contributing to airway remodeling in severe asthma 134. Fibroblast-EVs from scleroderma patients exhibited dysregulated collagen-related miRNA levels and further upregulated the expression of type I collagen in fibroblasts to facilitate wound healing 135. Similarly, fibroblast-EVs could shuttle miR-23a-3p to accelerate scratch closure of epidermal keratinocytes *in vitro* 136. Moreover, fibroblast-EVs significantly inhibited the production of ROS and cell death in fibroblasts induced by ultraviolet B radiation, thus playing a critical role in skin homeostasis during photoaging 137.

#### Adipocyte-derived EVs

Nowadays, the adipose tissue has been recognized as an endocrine organ that secretes pleiotropic bioactive molecules that modulate metabolism in distant organs and immune cell functions 138. Adipogenesis was thought to affect EV structure, molecular composition, and function, as EV production was higher in cells before adipogenesis 139. Interestingly, mouse perigonadal adipose tissue in leptin-deficient obese mice released more EVs than that of lean mice 140, and the cargoes and regulatory functions of these EVs were also different 141, 142. Protein profiling has revealed that EVs released by adipose tissue carried adipokines such as adiponectin, IL-6, monocyte chemoattractant protein-1, and resistin 143. In addition, these EVs also harbored enzymes including fatty acid synthase, acetyl-CoA carboxylase, glucose-6-phosphate dehydrogenase, immunomodulatory proteins and cytokines, as well as various mRNAs 144, 145. Further, two subpopulations of adipocyte-EVs, small EVs and large EVs, were identified and shown to exhibit specific protein signatures 146. Notably, the functions of EVs released from adipocytes in different differentiation states were varied. For instance, EVs from immature adipocytes were shown to induce telogen-to-anagen transitions in hair follicles, whereas those from mature adipocytes inhibited hair follicle progression 147. Therefore, it can be hypothesized that under different circumstances, adipocytes secrete EVs with distinct regulatory functions, contributing to diverse immune responses.

Adipocyte-EVs, now regarded as a new adipokine, exert regulatory functions in metabolic processes and insulin resistance 148. It was reported that circulating EVs were significantly increased in obese mice and humans compared to lean controls, and could be biomarkers of metabolic stress 149. Besides, EVs derived from the adipocytes of obese mice increased appetite and weight when administered to lean mice, whereas EVs from the adipocytes of lean mice decreased the weights of obese mice 150, indicating their regulatory role in obesity and metabolic diseases. Another study reported that obese adipocytes released high levels of exosomal miR-27a, which repressed PPAR-γ and induced insulin resistance in skeletal muscle 151.

In addition, adipocyte-EVs play pathogenic roles in immune responses via exerting effects on various cells. Adipocyte-EVs from obese mice induced M1 macrophage polarization to exacerbate intestinal inflammation 152. In addition, EVs from diabetic adipocytes or high glucose/high lipid-challenged non-diabetic adipocytes were enriched in miR-130b-3p, which aggravated myocardial ischemia/reperfusion injury in the diabetic heart 153. Similar findings have been reported in perivascular adipose tissues, where adipocyte-EVs from obese mice evoked inflammatory responses and vascular remodeling 154. Moreover, TNF-α/TNF-α^+^hypoxia-induced adipocyte-EVs enhanced neutrophil adhesion by increasing vascular cell adhesion molecule expression on one vascular cell line 155. Considering the critical roles of adipocytes in skin repair and inflammatory states, future studies of these EVs will have far-reaching implications.

## EV involvement in the pathophysiology of inflammatory skin disorders

Chronic inflammatory skin diseases, including psoriasis, AD, LP, and SLE, among others, are refractory with long-lasting courses. Though biological drugs such as anti-TNF, anti-IL-17, and anti-IL-12/23 agents show treatment-associated benefits, the complex pathogenesis is insufficiently understood, which warrants further exploration. Here, we will mainly describe how EVs participate in complicated pathophysiological processes involved in inflammatory skin disorders.

### Psoriasis

Psoriasis is the most common chronic inflammatory skin disease and is characterized by abnormal proliferation and differentiation of keratinocytes and massive infiltration of immune cells 156. A number of studies have shown that endothelial cell- and platelet-derived EVs were increased in patients with psoriasis 157-161, were positively correlated with the psoriasis area and severity index score 157, and were decreased by anti-TNF-αbut not anti-IL-12/23 treatment 162. Besides, psoriasis-related cytokines modulated the production of EVs, as IL-17A induced HaCaT cells to produce EVs carrying the mRNAs of several chemokines and β‐defensin 2 125. Recent studies attempted to analyze the miRNA profiles in plasma-derived EVs to illustrate their potential as future psoriasis biomarkers 17, 163.

**Figure 1:**
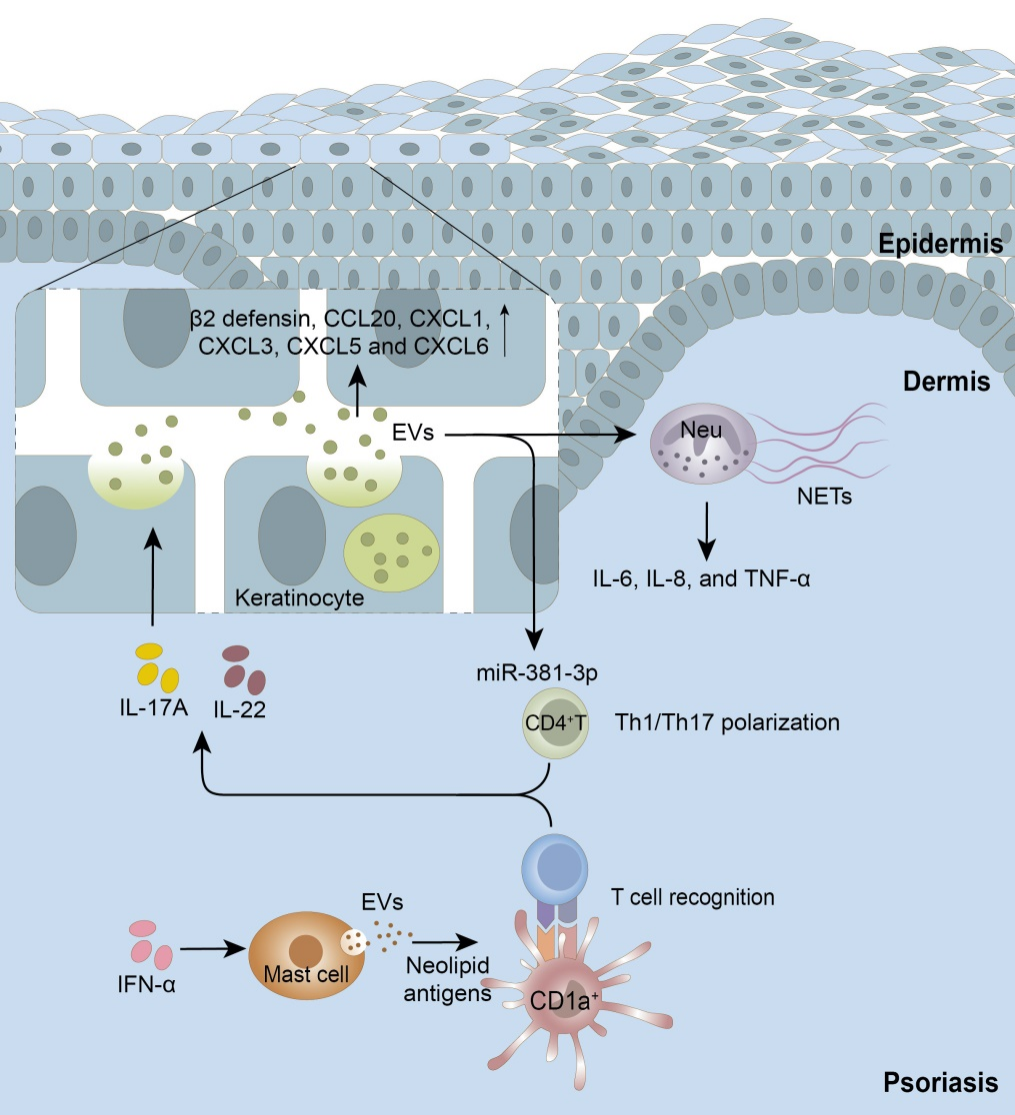
** Extracellular vesicles (EVs) in the cell-cell crosstalk of psoriasis.** Keratinocyte-EVs induced by psoriatic cytokines such as IL-17A can be taken up by neighboring keratinocytes to upregulate mRNA expression of β2 defensin and chemokines, by neutrophils to induce NETosis and the production of IL-6, IL-8, and TNF-α in neutrophils, or by CD4^+^T cells to facilitate Th1/Th17 polarization. In turn, neutrophil-EVs also induce keratinocytes to produce a variety of chemokines to attract more immune cells. In addition, IFN-α promotes mast cells to secrete EVs, which induces the generation of neolipid antigens and subsequent recognition by lipid-specific CD1a-reactive T cells. Neu, neutrophils; NETs, neutrophil extracellular traps.

Studies on the functions of EVs in psoriasis have gradually developed in recent years (**Figure 1**). As one early study revealed, IFN-α induced mast cells to release EVs that were capable of transferring cytoplasmic PLA2 activity to neighboring CD1a-expressing cells, which further led to the generation of neolipid antigens and subsequent recognition by CD1a-reactive T cells 11, which established EVs as critical mediators in psoriasis. Our recent study demonstrated that EVs isolated from psoriatic cytokine-induced keratinocytes could be endocytosed by neutrophils and induced the latter to produce NETs and pro-inflammatory cytokines, thus exacerbating psoriatic inflammation 12. Interestingly, neutrophils from patients with generalized pustular psoriasis secreted more EVs than those from controls, and further triggered keratinocytes to produce high levels of inflammatory molecules, such as IL-1β, IL-36G, IL-18 and TNF-α 82. These results suggest that EVs are critical mediators of keratinocyte-neutrophil crosstalk in the pathogenesis of psoriasis. Moreover, our latest study revealed that EVs derived from psoriatic keratinocytes transferred miR-381-3p to CD4^+^ T cells, inducing Th1/Th17 polarization and promoting psoriasis development 164. Interestingly, it was reported that EVs from two similar diseases, rheumatoid arthritis (RA) and psoriatic arthritis (PsA), showed divergent effects, as RA-derived or healthy control-derived EVs profoundly inhibited osteoclast differentiation while PsA-derived EVs had a stimulatory effect 165. Therefore, by comprehensively studying the biological functions of EVs, we will gain important insights regarding their roles in the immune network of psoriasis, or other associated syndromes.

### Atopic dermatitis

**Figure 2:**
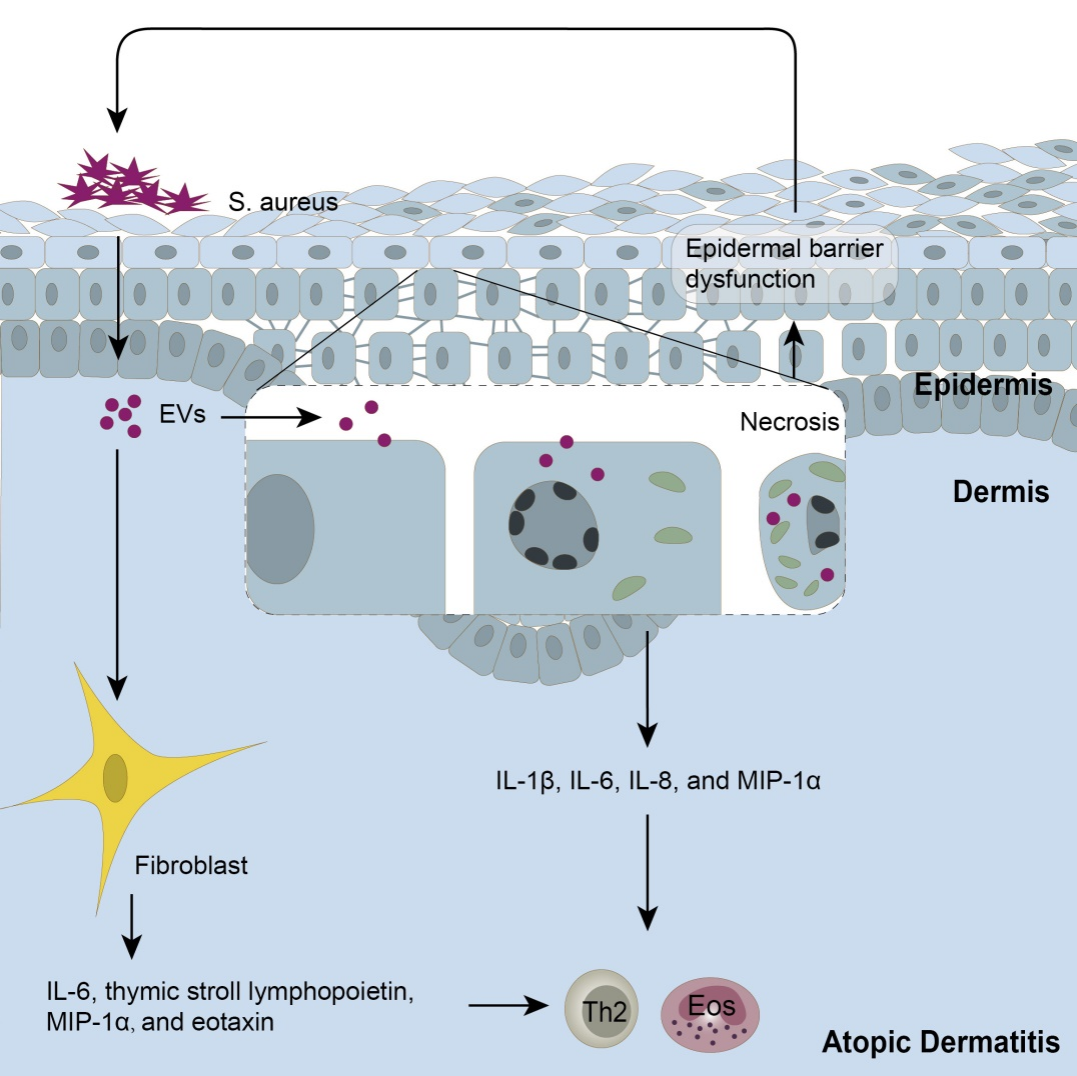
** Extracellular vesicles (EVs) in the pathogenesis of AD.** On the one hand, EVs secreted from *S. aureus* could induce keratinocyte necrosis and damage to epidermal barrier structures and functions, which in turn facilitates further colonization of *S. aureus*-EVs. These keratinocytes produce IL-1β, IL-6, IL-8, and MIP-1α in response to *S. aureus*-EVs. On the other hand, *S. aureus*-EVs upregulate the production of pro-inflammatory mediators in fibroblasts, including IL-6, thymic stromal lymphopoietin, macrophage inflammatory protein-1α, and eotaxin, contributing to the Th2 immune response in AD pathogenesis. *S. aureus*, *Staphylococcus aureus.*

AD is another common inflammatory skin disease, characterized by typical type 2 skin inflammation with a defective barrier 166, 167. As AD patients are susceptible to *S. aureus* infection, which in turn aggravates AD inflammation-associated, several studies have explored the role of *S. aureus*-derived EVs in AD. *S. aureus*-EVs could exacerbate AD inflammation by delivering bacterial effector molecules to host cells, thus aggravating the inflammatory responses (**Figure 2**). For instance, it was reported that *S. aureus*-EVs efficiently increased the production of pro-inflammatory mediators such as IL-6, thymic stromal lymphopoietin, and macrophage inflammatory protein-1α in dermal fibroblasts 168, triggered HaCaT cells to overexpress pro-inflammatory cytokines including IL-1β, IL-6, IL-8, and MIP-1α 169, and induced endothelial cell activation and monocyte recruitment 13. In addition, it was shown that α-hemolysin transported in *S. aureus*-EVs induced keratinocyte necrosis, exacerbating both skin barrier disruption and AD-like skin inflammation 170. Thus, *S. aureus*-EVs may be regarded as one of the therapeutic targets for the management of AD aggravation. Notably, EVs derived from thymol-treated *S. aureus* or *Lactobacillus plantarum* alleviated the AD-like skin lesions including epidermal thickening and IL-4 level 171, 172, indicating their potential to treat AD.

### Lichen planus

LP is the third most common inflammatory skin disease, characterized by epidermal keratinocyte death and dense infiltration of T cells in the dermis 173. We previously provided evidence of the role of IFN-γ in the cell-mediated cytotoxicity of keratinocytes in LP 174. Recently, several studies explored the function of EVs in LP. For instance, one study profiled salivary EVs from patients with oral LP and identified miR-4484, miR-1246, and miR-1290 as enriched miRNAs in LP, making these molecules potential biomarkers for oral LP 19. In addition, it was shown that circulating EVs in erosive oral LP patients enhanced T cell proliferation and attenuated T cell apoptosis 14. Thus, further studies are warranted to profile and determine the pathogenic roles of EVs derived from skin lesions or circulation of LP patients.

### Bullous pemphigoid

BP is a severe autoimmune inflammatory disorder and clinically manifests as subepidermal blisters and erosions of the skin and/or mucous membranes 175. Blister fluid contains infiltrated immune cells, cytokines, and chemokines, as well as EVs, all of which are capable of exerting biological functions and promoting inflammatory responses. We showed that EVs isolated from the blister fluid of BP patients could be internalized by human keratinocytes, which led to the production of critical inflammatory cytokines and chemokines, enhancing neutrophil trafficking to skin lesions and triggering local autoinflammatory responses 15. Neutrophils were reported to be critical for blister formation and exacerbated inflammation in BP 176, 177. We also employed mass spectrometry to elucidate the proteome of blister fluid-derived EVs for the first time, showing that they contained a variety of proteins implicated in autoimmunity and inflammatory responses 15. However, the contributions of neutrophil-, T cell-, B cell-, and other monocyte-EVs to the initiation and progression of BP are largely unknown.

### Systemic lupus erythematosus

SLE is a chronic, refractory, and systemic autoimmune disease characterized by circulating autoantibodies and the formation of immune complexes. It harms multiple organs and presents a variety of clinical manifestations 178. To date, the contents and functions of EVs carrying autoantigens, cytokines, surface receptors, and non-coding RNAs have been studied in SLE.

As early studies reported, the total number of EVs and IgG-positive EVs were increased in the plasma of SLE patients 18, 179, and their high levels were positively correlated with anti-DNA levels, suggesting that these EVs could represent an important source of immune complexes in SLE 180. Nielsen et al. showed that the level of annexin V non-binding cell-derived EVs was positively correlated with disease severity and some systemic indicators 181. They further found that the levels of IgG, IgM, and C1q were elevated in EVs from SLE patients compared with those from healthy controls 182. Nevertheless, it was not clearly demonstrated whether IgG in EVs had autoantibody activity. It was also reported that CD31^+^/annexin V^+^/CD42b^-^ MVs 182 or CD41^+^ EVs harboring IgG 183 were higher in SLE patients than in controls. Using high-sensitivity nano-liquid chromatography tandem mass spectrometry, 248 proteins were found to be significantly upregulated in the circulating EVs isolated from SLE patients relative to those of the controls 184. In addition, another recent study measured EVs by flow cytometry to identify small (0.2-0.7 μm) and large (0.7-3.0 μm) EVs, and showed that patients with active lupus nephritis had increased levels of large EVs containing mitochondria (mitoEVs) and IgG-positive mitoEVs, indicating that distinct EV subpopulations can have different functions in SLE 185. Further, urinary EVs of SLE patients were also identified and enriched in miRNAs, with miR-146a being the most prominent 186.

Moreover, it was shown that these increased EVs in the plasma of active SLE patients induced ROS production and degranulation in neutrophils 187, activated pDCs to secrete IFN-α via TLR7 188, or contributed to MSC senescence in SLE 189. The elevated EVs and their immune complexes in SLE patients also promoted secondary leukocyte infiltration by regulating vascular remodeling and chemokine secretion 16. Moreover, EVs derived from platelets mostly harbored IgG and overexpressed CD69 and CD64, and promoted pro-inflammatory responses in monocytes, thus exacerbating SLE-associated inflammation 190. Thus, it is worth noting that EVs play a crucial role in initiating and aggregating autoimmune reactions in SLE.

### Chronic wound healing

Wound healing is a complex and dynamic process that includes hemostasis, inflammation, angiogenesis, re-epithelialization and remodeling 191. However, the disturbances in this process, common in diabetes and aging individuals, lead to chronic wounds, a dilemma in dermatology 192, 193. A variety of cell types with distinct roles are involved in the different phases of chronic wound healing, including neutrophils 194, macrophages 195, mast cells 196, DCs 197, T cells 198, fibroblasts 199, and others 192. Therefore, EVs, cytokines, growth factors, and chemokines derived from multiple sources can actively regulate complex cellular signaling networks.

Numerous studies have focused on pathogenic factors and new techniques to promote wound healing. According to recent studies, EVs can contribute to multiple stages of wound healing cascades such as cell proliferation and differentiation 200, coagulation 201, angiogenesis 202, and extracellular matrix remodeling 203. For instance, EVs isolated from platelet-rich plasma could effectively induce the proliferation and migration of endothelial cells and fibroblasts to improve angiogenesis and re-epithelialization in chronic wounds, therefore showing efficacy in chronic wounds in a diabetic rat model 204. EVs released from corneal epithelial cells were enriched in provisional matrix proteins, fibronectin, and thrombospondin-1, promoting the differentiation of myofibroblasts in the development of corneal scars 200. Similarly, EVs carrying miR-21, which were mainly derived from resident keratinocytes, were elevated in the wound fluid of healing chronic wound patients, and able to convert M1-polarized human macrophages to fibroblast-like cells; however, the conversion was strikingly impaired in a mouse model of experimental diabetes, and could be rescued by nanoparticles delivering miR-21 to macrophages 205. However, advanced glycation end products induced human umbilical vein endothelial cells to secrete EVs enriched in miR-106b-5p, which triggered fibroblast autophagy, thus decreasing collagen synthesis and delaying cutaneous wound healing 206. EVs derived from normal and diabetic human corneolimbal keratocytes exhibited distinct contents, and accelerated or delayed the proliferation of limbal epithelial cells in wound healing 207. Therefore, we hypothesize that EVs derived from various sources contribute to the local immune responses in chronic wound healing.

## Therapeutic approaches of EVs in inflammatory skin disorders

### EVs as biomarkers in inflammatory skin disorders

EVs are mainly regarded as diagnostic and prognostic biomarkers in cancer but are still largely unexplored in skin inflammatory diseases. Recently, increasing evidence has indicated their potential role as noninvasive biomarkers for predicting the onset, relapse or reaction to drugs in the field of inflammatory skin disorders.

**Figure 3:**
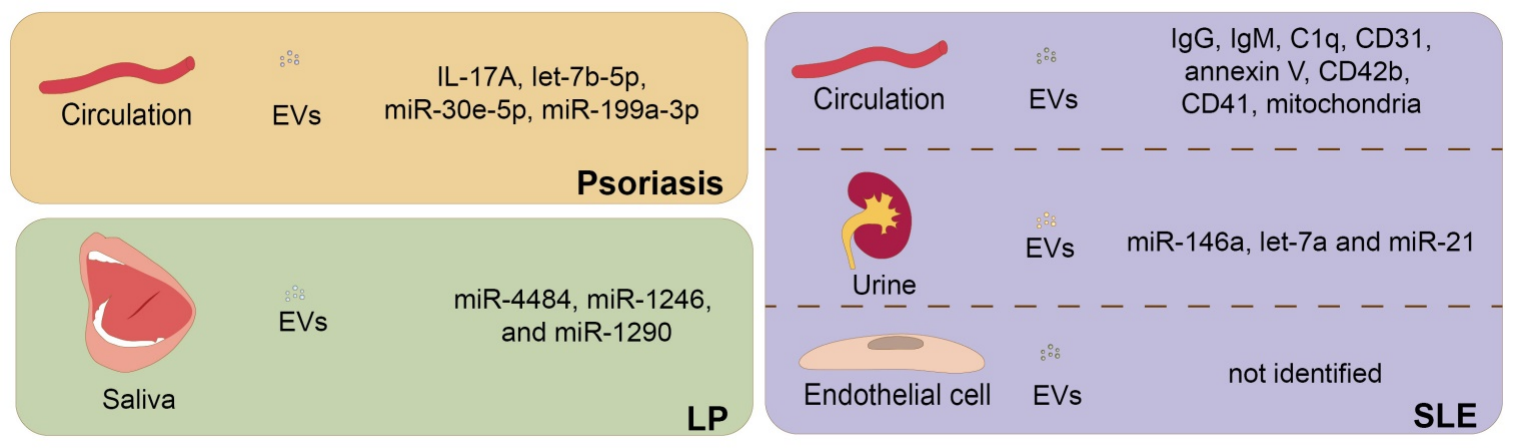
** Extracellular vesicles (EVs) are potential biomarkers in inflammatory skin disorders.** EVs derived from various origins have the potential to serve as biomarkers in several inflammatory dermatoses, including psoriasis, LP, and SLE. In psoriasis, the level of IL-17A^+^ EVs in circulation was significantly higher in patients with moderate-to-severe psoriasis than in those with mild psoriasis. MiR-199a-3p was the most up-regulated in serum EVs from psoriasis patients, implicating its diagnostic role in future study. Let-7b-5p and miR-30e-5p in serum EVs were significantly lower in patients with PsA, suggesting that circulating EV miRNAs might serve as biomarkers for PsA. In SLE, the increase in endothelial EVs or circulating EVs carrying several immune molecules was positively correlated with disease activity. And urinary EVs of SLE patients were also identified and enriched in miRNAs, with miR-146a being the most prominent. Urinary EVs containing let-7a and miR-21 were significantly down-regulated in lupus nephritis patients, and elevated after the complete course of effective treatment. In LP, salivary EVs of patients from oral LP were enriched in miR-4484, miR-1246, and miR-1290, making them potential biomarkers for oral LP.

Several studies on psoriasis, SLE, AD, and LP 19 aimed to explore the role of EVs as biomarkers (**Figure 3**). For instance, the level of IL-17A^+^ EVs in circulation was significantly higher in patients with moderate-to-severe psoriasis than in those with mild psoriasis, suggesting that the components of EVs could be indicators of distinct disease stages 161. Another study further compared the plasma-derived EV miRNAs from cutaneous-only psoriasis patients (n=15) with those of PsA patients (n=14), showing that let-7b-5p and miR-30e-5p in plasma-derived EVs were significantly lower in PsA patients, which suggested that circulating EV miRNAs might serve as biomarkers for PsA 163. A recent study profiled the miRNAs of EVs from psoriasis patients (n=52) and healthy controls (n=26) and revealed that 26 miRNAs were upregulated and 24 were downregulated, with miR-199a-3p the most differentially expressed in psoriasis 17. These results highlight the potential of EVs as diagnostic markers for psoriasis patients, however, larger samples and the pathogenic roles of EVs should be considered in the future. In SLE, several studies have suggested that the levels of circulating EVs were correlated with disease activity and clinical features, indicating that EVs could be reliable biomarkers of SLE activity 18, 181, 184. For instance, it was shown that the increase in endothelial EVs was positively correlated with disease activity, glomerulonephritis, and vascular dysfunction 181, and was reduced by immunosuppressive therapy with decreasing cardiovascular risk 208. Urinary EVs containing let-7a and miR-21 were significantly down-regulated in lupus nephritis patients and elevated after complete course of effective treatment, suggesting that urinary EV-associated miRNAs could be used as liquid biopsies to estimate the clinical stage of lupus nephritis patients 209. In addition, as one recent study preliminarily explored, serum microbial EVs showed potential as novel biomarkers for AD diagnosis 210.

Further studies of the novel ways that EVs can reflect disease pathogenesis or clinical stages, predict relapse or prognosis, indicate responses to therapies, or guide therapy beyond current biomarkers are needed.

### EVs as therapeutic agents in inflammatory skin disorders

Currently, EVs are being explored as nanotherapeutic agents for immune therapy, regenerative medicine, and drug delivery. In dermatology, studies on EVs are just beginning to show promising prospects in inflammatory skin disorders. As mentioned above, adipose tissue-derived MSC-EVs showed promising results as a cell-free therapeutic modality for AD treatment 123. Several other investigations have demonstrated the potential uses of MSC- and APC-derived EVs as cell-free agents to treat autoimmune diseases. It was reported that the transplantation of MSCs would rescue bone marrow MSC function in a lupus knock-out mouse model via transferring EVs-derived Fas to recipient cells 211. Another study showed that MSC-EVs were successfully used to treat a patient with refractory graft-versus-host disease and showed sustained alleviation of cutaneous and mucosal manifestations after 4 months 212.

Moreover, several studies have shown that EVs can be used as therapeutic agents in wound healing. EVs derived from umbilical cord blood were shown to be enriched in miR-21-3p, and promoted the proliferation and migration of fibroblasts and enhanced the angiogenic activities of endothelial cells, thus accelerating re-epithelialization and cutaneous wound healing 213. Keratinocyte-EVs could also modulate fibroblast function and angiogenesis by transferring miR-21 to facilitate skin wound healing 7. EVs from oral mucosa epithelial cells of human healthy donors showed pro-regenerative effects on skin wound healing 214, and EVs from human urine-derived stem cells could promote angiogenesis and wound healing in diabetic mice 215. Interestingly, *Synechococcus elongatus*-EVs were capable of augmenting endothelial angiogenesis and stimulating new blood vessel formation, indicating that *Synechococcus elongatus*-EVs maybe a promising strategy for wound healing 202. It was shown that MSCs-EVs maintained or accelerated rapid wound healing by activating fibroblasts 203, epithelial cells 216, or gingiva wound healing 217, which provided a potential therapeutic approach in this field.

Nowadays, the combination of EVs and biomaterials to enhance the wound healing process represents a novel approach for chronic wound therapy 218. One study developed an injectable antibacterial hydrogel with stimuli-responsive adipose-derived MSC-EVs, which could efficiently accelerate chronic wound healing and skin regeneration with coordinated actions 219. More recently, a study reported that a light-activated hydrogel containing small EVs isolated from human mononuclear cells promoted wound healing, a process that was partially orchestrated by EV-miRNAs 220. Additionally, allogenic EVs isolated from adipose tissue-derived stromal cells were engineered into a thermoresponsive gel, which resulted in a statistically significant improvement in fistula healing 221. To date, several phase-I or phase-II clinical trials have been conducted to study whether EVs can be used in humans without life-threatening complications 222, 223. However, only one clinical trial was established to study the use of EVs in the treatment of skin diseases. AGLE-102 is an allogeneic EV derived from normal donor MSCs. One phase 1/2A, multi-center, randomized study aimed to assess the safety and efficacy of AGLE-102 in the treatment of lesions in epidermolysis bullosa patients (NCT04173650).

Importantly, EVs, especially exosomes, have great potential as drug delivery vehicles. They are suitable for delivering therapeutic agents due to their natural properties, including material transportation, stability in circulation, relatively long half-lives, and excellent biocompatibility 224. For instance, EV-based drug formulations offer a powerful and novel delivery platform for anti-cancer and -inflammation therapy 225-227. For example, since the transcription factor nuclear factor kappa-light-chain-enhancer of activated B cells (NF-κB) plays a pivotal role in modulating the inflammatory cascades in a variety of inflammatory diseases, including septic shock and psoriasis, an optogenetically engineered EV system was employed to load a large amount of super-repressor IκB into EVs. These engineered EVs were taken up by recipient cells and then attenuated systemic inflammation in septic model mice 228. Though several techniques are in use for drug loading and targeted delivery, a standard effective method is still lacking. Existing methodologies include the incubation of drugs with EVs, electroporation or saponification to induce the formation of small pores within the membranes, freeze-thaw methods that can cause the degradation of many EV proteins, and the transfection of nucleic acids into the secreting cells, among others 229. Any method can be selected depending on the study demand and its advantages. Therefore, to exploit the therapeutic potential of EVs, innovative approaches and full considerations are required.

Based on the availability, cost of production and safety, plants, fruits, and even milk can be used as sources from which to isolate EVs for clinical use 230. For instance, anti-tumor drugs such as doxorubicin and curcumin were loaded on grapefruit-EVs decorated with targeting modifications, and showed anti-inflammatory effects in tumor-bearing mice 231. Milk-EVs were reported to survive harsh and degrading conditions in the gut and then taken up by various cell types. Thus, several studies isolated EVs from milk and attempted to load them with various drugs with high yield and safety 232-234. Thus, these innovations and updated techniques can be considered in the future study of EV utility in inflammatory skin diseases.

In dermatology, the combination of EVs and biomaterials in regenerative medicine warrants in-depth study in mouse models and clinical trials, for instance, in wound healing and hair regeneration. The topical use of this kind of EV will also show promising prospects in treating inflammatory skin diseases. With improved understanding of EV functions and the development of related bioengineering strategies, it is possible to develop therapeutic applications such as EVs enriched in the cargo of interest, with enhanced targeting properties or decorated with physiological or synthetic ligands to target specific receptors that are overexpressed in different autoimmune diseases.

## Challenges and Future Directions

Currently, several major challenges limit the broader translational use of EVs. As the EV classifications are continuously evolving, one problem is the lack of unique markers for the heterogeneous subclasses of EVs, which overlap in sizes, biophysical properties, and contents 235. Therefore, the ISEV suggested the use of physical characteristics (such as small EVs), biological composition (some protein positive EVs), or descriptions of conditions (such as hypoxic EVs) to name the EV subtypes 236. Since the isolation methods and approaches for EV analysis are evolving 237, standard methods need further validation. One proposed solution is to comprehensively study the biogenesis and cargo content of all EVs from multiple cell types 238. Using asymmetric flow field-flow fractionation, one group identified two exosome subpopulations, large exosomes (90-120 nm) and small exosomes (60-80 nm), and discovered an abundant population of non-membranous nanoparticles termed “exomeres” (~35 nm). Each subpopulation showed unique protein and nucleic acid profiles, as well as biophysical properties, suggesting a distinct biological function for each subpopulation 239. Besides, fourier-transform infrared spectroscopy provided collective fingerprints of EV subpopulations including large (~600 nm), medium (~200 nm), and small (~60 nm) EVs 240. Moreover, diverse subpopulations of plasma EVs were identified using high-sensitivity fluorescence-activated vesicle sorting and showed that erythrocyte- and platelet-derived EVs carried distinct repertoires of nucleic acids that were similar to those of their original sources 241. Therefore, as increasing evidence has shown that EV subclasses carry different cargoes, it is necessary to develop standardized and effective methods to identify and isolate the different EV subpopulations.

Current EV isolation methods yield many non-vesicular proteins and contaminated EVs, which limit the profiling of nucleic acids or proteins in EVs. Thus, several techniques were developed to improve our understanding of EV contents. A recent study used a trypsin-digested proteomics approach to classify trypsin-sensitive and trypsin-resistant vesicular proteins, and to systematically study the actual-vesicular proteins, which helped to identify reliable functional proteins, and revealed their pathophysiological roles, an important aspect in the use of EVs as liquid biopsies 242. In addition, new techniques such as exosome-templated nanoplasmonics 243 and nanopatterned microchips 244 were recently developed to accurately and sensitively monitor the molecular profiles of EVs, which would facilitate the development of liquid biopsies.

Further, how to identify and clearly observe EVs has been a major obstacle in understanding EVs and their utility. Notably, new technologies have flourished in recent years. For instance, one study used a non-pH-sensitive red fluorescent tag to visualize the lifecycle and biogenesis of EVs, including multivesicular body (MVB) trafficking, MVB fusion, EV uptake, and endosome acidification 245. To quantify EV uptake at the single-cell level, another study developed an engineering approach that combined mass cytometry with highly multivariate cellular phenotyping. This innovation could help to unravel the *in vivo* fate of EVs taken up by recipient cells, elucidating the mechanism of action of EVs* in vivo* 246. The updated technologies tracking EVs will improve our mechanistic understanding of the biodistribution of EVs.

Nowadays, efforts to increase the production of EVs by cells for future therapeutic applications are ongoing. Conventional isolation methods such as ultracentrifugation require multiple steps that cause significant loss and damages to EVs. To overcome this limitation, researchers have developed several methods to increase the yield of EVs or to engineer EVs. One strategy is to increase EV biogenesis in the donor cell by overexpressing regulatory proteins involved in EV biogenesis 247 or increasing MVB docking at the plasma membrane 248. Besides, physical conditions including radiation, hypoxia, and low pH were reported to upregulate EV production. For instance, nitrogen cavitation was used to instantly disrupt neutrophil-like HL60 cells leading to a 16-fold increase in the formation of nanosized membrane vesicles, which were then loaded with an anti-inflammation drug to treat acute lung inflammation/injury and sepsis induced by LPS 249. Ultrasonication of MSCs for 1 min also improved the EV yield, which exhibited ∼20-fold higher and ∼100-fold faster production than the natural secretion 250. A recent study described a detailed method to fabricate an origami-paper-based device that integrated the ion concentration polarization technique for effective enrichment and isolation of EVs in a simple manner 251. Further, engineered EVs showed promising roles in this field. One group developed a system to engineer EVs to sustainably release TGF-β1, IL-2, and rapamycin to induce Treg differentiation from naïve T cells 252. However, more studies should be conducted to ensure that the engineered EVs exhibit stable bioactivities and efficacies, stable packing of biological materials, and little cytotoxicity.

## Conclusions

In recent years, we have witnessed a boom in EV studies in the context of nearly all diseases, such as in the dysregulation and treatment of cancers, inflammation, and autoimmune diseases. Therefore, we reviewed the regulatory functions of immune and non-immune cell-derived EVs, and their roles in inflammatory skin disorders, as triggers of immune responses, potential biomarkers, or therapeutic agents. However, the studies are just beginning in dermatology, and the precise contents of most EV preparations or their multiple functions remain to be deciphered. Likewise, new strategies and more comprehensive studies are needed to identify EV subpopulations with high accuracy and selectivity, and to address the emerging challenges. We are optimistic that continuing studies on EVs will allow future application of EVs in the detection and treatment of various inflammatory skin diseases.

## Figures and Tables

**Table 1:** Functions of EVs derived from immune or nonimmune cells

Source cell	Contents (native)	Contents (modified)	Target cell	Effects	Ref.
**Immune cell-derived EVs**					
DC-EVs	MHC-I and -II, T-cell costimulatory molecules, ICAM-1, MHC-peptide complexes.		DCs	Further initiate the alloreactive T cell responses and acute rejection (*in vivo*)	29
			T cells	Promote Th1/Th2 cytokine secretion	26-28, 32
			Resident DCs.	Further activate CD4^+^/CD8^+^T cells.	23
	TLR4		BMDCs	Increase cellular responsiveness to LPS (*in vivo*).	33
	Circulating antigens and allergens		Mast cells	Induce mast cells to degranulate and trigger anaphylaxis	34
		Multiple RNA classes	T cells	Balance Th1/Th2 responses	24, 25
		Viral components	T cells or other cells	Transfer infectivity in HCV, Dengue virus, and HIV infection.	35-37
B cell-EVs	MHC-I and -II, costimulatory and adhesion molecules, CD20, CD45, heat shock proteins, and pyruvate kinase type M2.		DCs and others	Promote antigen presentation and T cell responses	39, 40, 42
	C3 fragments		T cells		41
	FasL		Autologous CD4^+^ T cells	Induce antigen-specific apoptosis	46
		Anti-miR-155	Macrophages	Down-modulate endogenous miRNA	45
T cell-EVs	MHC-I and -II, TCR, CD3, APO2 ligand, adhesion molecules, FasL, and chemokine receptors.		Various	Exert immune regulatory effects, such as inhibiting NK cytotoxicity, regulating DC maturation, and enhancing B cell responses and promoting antibody production.	4, 49-53
		CD8^+^T cell-EVs	Mesenchymal tumor stromal cells	Prevent tumor progression	56
	MiRNAs or lipids		B cells	Modulate B cell survival, proliferation, and antibody production	4, 53, 58
			Th1 cells	Inhibit the proliferation of Th1 cells and IFN-γ secretion	55
	MiRNAs		Various	Implicate distinct functions of T cell subsets-derived EVs	54
	Not specific		Mast cells	Induce mast cells to degranulate and release several cytokines	57
Macrophage-EVs	Functional proteins		Various	Exert pro-inflammatory role in inflammation	60-62
	Cholesterol			Lower the cholesterol level	63
	Wnt proteins		Dermal papilla cells	Enhance the hair follicle growth	64
	MiRs		Monocytes	Induce naïve monocyte differentiation	65
		Not specific	Neutrophils	Promote ROS production and subsequent necroptosis	66
	Not specific		Fibroblasts, hepatocyte, or epithelial cells, etc.	Contribute to a variety of inflammation and tissue injures, and regulates endothelial cell migration	67-69, 72
	Not specific		Human umbilical vein endothelial cells	Inhibit inflammation and accelerate diabetic wound healing (*in vivo*)	71
Neutrophil-EVs	Neutrophil-associated receptors, granule proteins, annexin A1.		Various	Modulate the pro-inflammatory or anti-inflammatory responses of target cells	77-84
			Vascular endothelial cells.	Modulate endothelial permeability and vascular remodeling.	81, 99-104
	Arachidonic acid		Platelets	Promote platelet-mediated innate immune responses	87
		Phosphatidylserine (PS)	Various	Suppress C5a priming of the inflammasome activation (*in vivo*)	98
	Antimicrobial proteins		Bacteria or fungus	Anti-infections	76, 86
		Mycobacterium tuberculosis-infected neutrophil-EVs.	Macrophage	Induce autophagy and superoxide anion production in macrophage, thus indirectly promoting the clearance of intracellular mycobacterium tuberculosis.	88
	MiRNAs and lncRNAs.			Implicate roles in diagnosis and therapeutics in autoimmunity.	85
**Nonimmune cell-derived EVs**					
Mesenchymal stem cell-EVs	Functional proteins		Various	Regulate angiogenesis, apoptosis, inflammation, proteolysis, and extracellular matrix remodeling	106-112
	Distinct classes of RNAs				
	Pro-angiogenic transcription factors and others		Tubular epithelial cells.	Regulate proliferative or anti-apoptotic pathways	115
				Suppress CX3CL1 expression	116
				Promote the recovery of kidney function (*in vivo*).	117
	Not specific		Various cells in liver diseases.	Suppress the epithelial-to-mesenchymal transition- hepatocyte regeneration	119
				Increase hepatocyte regeneration	119
				Decrease proliferation of hepatic stellate cells	120
				Reduce the level of serum alanine aminotransferase and pro-inflammatory cytokines (*in vivo*).	121
	Not specific		Various	Ameliorate the atopic dermatitis inflammation (*in vivo*).	123
Keratinocyte-EVs		MHC molecules	T cells	Promote CD4^+^ and CD8^+^ T cell proliferation	126
		Antigens	BMDCs	Help BMDCs to differentiate into mature phenotype and produce large amounts of IL-6, IL-10 and IL-12	131
		β-defensin 2 and chemoattractants	Various	Amplify the pro-inflammatory cascade	125
	Functional proteins		Fibroblasts	Modulate wound healing	128, 129
	MiRNAs			Help discriminate between EV subpopulations	127
		MiR-381-3p	CD4^+^ T cells	Induce Th1/Th17 polarization in psoriasis.	164
		MiR-203	Melanocytes	Regulate melanin synthesis by melanocytes in skin pigmentation	130
	Not specific		Keratinocytes	Suppress cell proliferation	6
			Carcinoma cell line TR146		
Fibroblast-EVs		Dysregulated collagen-related miRNA	Fibroblasts	Facilitate wound healing	135
	MiR-21-3p		Cardiomyocytes	Induce cardiomyocyte hypertrophy	133
	MiR-23a-3p		Keratinocytes/ epithelial cells	Accelerate cell proliferation and scratch closure	136
	Not specific		Fibroblasts	Protect cells against UVB-induced cell death	137
Adipocyte-EVs	Adipokines, Enzymes, immunomodulatory proteins and cytokines, and various mRNAs		Various	Exert regulatory effects on metabolic process and insulin resistance	143-145, 149, 150
	MiR-27a		Skeletal muscle cells	Induce insulin resistance	151
	MiR-155		Macrophage	Induce M1 macrophage polarization to exacerbate the intestine inflammation (*in vivo*)	152, 253
		MiR-130b-3p	Cardiomyocytes	Exacerbate myocardial ischemia/reperfusion injury	153
	Circ_0075932		Keratinocytes	Promote inflammation and apoptosis	141
		Not specific	Vascular cells	Evoke inflammatory responses and vascular remodeling, and increase vascular cell adhesion molecule expression	154, 155
	Not specific		Hair-compositing cells	Modulate hair follicle progression	147

The contents do not include the common identification markers of EVs; Abbreviations: BMDCs, bone marrow-derived dendritic cells; DCs: dendritic cells; ICAM-1, intercellular cell adhesion molecule-1; LPS: lipopolysaccharides; MHC: major histocompatibility complex; miR: microRNA; MSCs: mesenchymal stem cells; NK: natural killer; TCR: T cell receptor; Th: T helper; TLR4: Toll-like receptor 4; PD-L1, programmed death-ligand 1.

## Abbreviations

AD: atopic dermatitis
APCs: antigen presenting cells
BCR: B cell receptor
BP: bullous pemphigoid
DCs: dendritic cells
ECM: extracellular matrix
EV: extracellular vesicle
IFN-γ: interferon-γ
IgG: immunoglobulin G
ISEV: international society of extracellular vesicles
lncRNA: long non-coding RNA
LP: lichen planus
LPS: lipopolysaccharides
MHC: major histocompatibility complex
miR: microRNA
MSCs: mesenchymal stem cells
MVB: multivesicular body
MVs: microvesicles
NETs: neutrophil extracellular traps
NK: natural killer
RNA-seq: RNA-sequencing
S. aureus: staphylococcus aureus
SLE: systemic lupus erythematosus
TAMs: tumor-associated macrophages
TCR: T cell receptor
TEM: transmission electron microscope
TGF-β: transforming growth factor-β
Th: T helper
TLR4: Toll-like receptor 4
